# **Synthesis and evaluation of multi-wall carbon nanotube–paclitaxel complex as an anti-cancer agent**

**Published:** 2016

**Authors:** Fariba Ghasemvand, Esmaeil Biazar, Sara Tavakolifard, Mohammad Khaledian, Saeid Rahmanzadeh, Daruosh Momenzadeh, Roshanak Afroosheh, Faezeh Zarkalami, Marjan Shabannezhad, Saeed Hesami Tackallou, Nilofar Massoudi, Saeed Heidari Keshel

**Affiliations:** 1 *Enzyme Technology Lab, Genetics & Metabolism Research Group, Pasteur Institute of Iran, Tehran, Iran*; 2 *Department of Biomaterials Engineering, Tonekabon Branch, Islamic Azad University, Tonekabon, Iran *; 3 *Department of Chemistry, Science and Research Branch, Islamic Azad University, Yazd, Iran*; 4 *Brain and Spinal Injury Research Center, Tehran University of Medical Sciences, Tehran, Iran*; 5 *Department of Biology, Garmsar Branch, Islamic Azad University, Garmsar, Iran*; 6 *Department of Anesthesiology, Faculty of Medicine, Shahid Beheshti University of Medical Sciences, Tehran, Iran *; 7 *Proteomics Research center, Shahid Beheshti University of medical sciences, Tehran, Iran*; 8 *Stem cell preparation unit, Eye Research center, Farabi Eye Hospital, Tehran University of medical sciences, Tehran, Iran*

**Keywords:** Multi-walled carbon nanotubes, Paclitaxel, Structural investigations, Gastric cancer, MKN-45 cell line

## Abstract

**Aim:**

The aim of this study was to design multi-walled carbon nanotubes (MWCNTs) loaded with paclitaxel (PTX) anti-cancer drug and investigate its anti-cancerous efficacy of human gastric cancer.

**Background:**

Carbon nanotubes (CNTs) represent a novel nano-materials applied in various fields such as drug delivery due to their unique chemical properties and high drug loading.

**Patients and methods:**

In this study, multi-walled carbon nanotubes (MWCNTs) pre-functionalized covalently with a paclitaxel (PTX) as an anti-cancer drug and evaluated by different analyses including, scanning electron microscope (SEM), particle size analyzer and cellular analyses.

**Results:**

A well conjugated of anti-cancer drug on the carbon nanotube surfaces was shown. This study demonstrates that the MWCN-PTX complex is a potentially useful system for delivery of anti-cancer drugs. The flow cytometry, CFU and MTT assay results have disclosed that MWCNT/PTXs might promote apoptosis in MKN-45 gastric adenocarcinoma cell line.

**Conclusion:**

According to results, our simple method can be designed a candidate material for chemotherapy. It has presented a few bio-related applications including, their successful use as a nano-carriers for drug transport.

## Introduction

 The nanomaterials and their surface modification are applied for biomaterials devices such as tissue engineering, dental materials and drug delivery (1-14). Carbon nanotubes (CNTs) are probably the most wonderful member of the fullerenes family. Fullerenes comprise any molecule made entirely of carbon atoms, shaped as a sphere, an ellipsoid, or a tube (15-17). A high aspect ratio and high surface areas via many dangling bonds on the side walls of them evolves in MWNTs, having the potential to be modiﬁed with many different molecules, including: proteins, enzymes, nucleic acids, as well as drugs for chemical, biological, and medical applications (18-22). In recent years, significant benefits of nano-medicines can be the specific binding of drugs to the targeted cancer cells has been developed of novel drug delivery systems. The SWCNTs have been loaded with drug molecules such as doxorubicin (DOX) and paclitaxel (PTX) via π-π interactions. The rate of drug releasing has been shown to be controllable by nanotubes with various diameters (23). In patients with cancer (in cancerous diseases), carbon nanotubes have crucial roles in delivering pharmacological agents, as diagnostic imaging agents, oligonucleotides, and proteins to better dose scheduling for compliance of improved patients or treat a malignant tumour (23-25). The application of functionalized carbon nanotubes as new nano-vectors for drug delivering was apparent immediately after the first demonstration of the capacity of these materials to penetrate into cells. Thus, the great superiority of nanomedicines against conventional medicines is their potential to deliver drugs into tumors due to the leaky tumor vasculature, which provides better tissue penetration (26, 27). The innate properties of modified MWCNTs to treat several types of cancer result in minimal or no poisonous effects on normal cells. In this study, we describe a system based on paclitaxel- succinic anhydride, modiﬁed MWCNTs. These complexes were inquired by scanning electron microscope (SEM), Particle size analyzer (PSA), cellular analyses. 

## Patients and Methods

**Conjugation of Paclitaxel onto amid Functionalized MWNTs**

A 2′ hemisuccinate derivative (PTX-Suc) of PTX was prepared by the previously reported method with some modiﬁcations (19). Brieﬂy, paclitaxel (200 mg, 0.2 mmol) (C47H51NO14, PTX, Xi'an Natural-field Bio-technique Co., Ltd, China)) and succinic anhydride (0.2 mmol) was dissolved in CH_2_Cl_2_ (15 mL) at ambient temperature. Afterwards, pyridine (0.001 mmol) was added, the mixture was vigorously stirred for 3 days at ambient temperature. The reaction mixture was concentrated in vacuum and was purified by silica gel column chromatography with chloroform-methanol mixture (97:3). The puriﬁed PTX-Suc was obtained as a white solid. Covalently functionalized MWCNTs (MWCNT, purity, >95%, OD : 8-15 nm ; DI: 3-5 nm, Hengqiu, China) were prepared by dissolving PTX-Suc (100 mg, 0.1 mmol) in dimethyl sulfoxide (DMSO, Aldrich) was activated by N-hydroxysuccinimide (0.1 mmol, 0.011g) (C_4_H_5_NO_3_, NHS, Merck, UK) and 1-ethyl-3-(3-dimethylaminopropyl carbodiimide hydrochloride) (0.1 mmol, 0.019g) (C_8_H_17_N_3_, EDC.HCl, Fluka, USA) for 4h at room temperature to afford an NHS ester form of PTX-Suc. Subsequently, the produced mixture was added to MWCNTs, amide functionalized (10 mg,) (Sigma-Aldrich, Germany) in PBS (pH 7) and the reaction proceeded at 50°C for 24 h, Unbound excesses PTX-Suc were removed by ﬁltration and washed thoroughly with distilled water (over 10 times) and PBS until the ﬁltrate became free of white color (corresponding to free PTX-Suc). In the end, SEM measurement was carried out by the electron microscope (VEGA, TESCAN, Czech) and dynamic light scattering (DLS) was used in order to examine the size and size distribution of the prepared particles (Malvern, ZS3600, England). 

**Cell analysis and anti tumor activity**

In order to evaluate the cytotoxicity effect of components on the MKN-45cell line (National Cell Bank of Iran, Pasteur Institute), the 3-(4,5-dimethyl-2-thiazolyl)-2,5-diphenyl-2H-tetrazolium bromide (MTT) (Sigma-Aldrich, Germany) colorimetric assay was applied. The samples and negative control (Tissue culture poly styrene; TCPS) and positive control (paclitaxel anticancer powder; C47H51NO14, PTX, Xi' a Natural Field Bio-technique Co., Ltd, P. R. China) were well cleaned and sterilized by the autoclave method. Following that, the individual samples were placed in Petri dishes (Grainer, USA) using a sterilized pincer, cell culture MKN-45 gastric adenocarcinoma were cultured in RPMI 1640 supplemented with 10% fetal calf serum, 100 U/ml penicillin and 100 µg/ml streptomycin (all from Gibco, UK). Then, these samples were incubated at 37°C in a humidified CO2 incubator (Binder Company, Germany) with 5% CO2 and 95% air; in addition, Cultures were examined regularly. After that, MKN-45 cells were seeded (10000 cells per well) into 96-wells, flat-bottom microtiter plates (Grainer, USA) and incubated for 4h prior to the addition of filtered 5 different concentrations of the studied compounds. Final concentrations achieved in treated wells were 5, 10, 20, 35, 50 µM/ml. Furthermore, the optical density (OD) value was defined as the absorbance of each individual well. Finally, the absorbance was measured at 575 nm (test wavelength) and with a reference filter of 630 nm. All inquiries were performed three times and the viability was calculated and showed in figure 3E at the 24, 48, 72 and 96 h. The results of experiments were recorded as percentage absorbance relative to untreated control cells; additionally, the percentage of cell viability was calculated using the equation: (mean optical density (OD) of treated cells/mean OD of control cells) ×100. Thus, as to colony formation assay, MKN-45 cells were seeded in 3.5-cm dishes (1000 cells/dish); then, cultured for 2 weeks to allow for colony formation. In the next stage, after a period of 14 days the plates were stained with 3% crystal violet (Sigma, Germany) in methanol (Merck, Germany) at room temperature for 10 minutes and all visible colonies were counted. This step was repeated 10 times, and the data were presented as a mean standard deviation.

For cell cycle and apoptosis analysis, the cells were subjected with MWCNT, MWCNT-PTX, PTX, then removed, incubated and washed in cold PBS. The cells were re-centrifuged (Sigma, Germany) and discarded supernatant, then cells re-suspend in 1X annexin-binding buffer (0.1 M HEPES, pH 7.4; 1.4 M NaCl; 25 mM CaCl 2. Dilute to 1X prior to use; BD, USA). In determining the cell density, they were diluted in 1X annexin-binding buffer to ~1 × 10^6^ cells/mL (Gently mix the cells and incubate for 15 min at RT in the dark), to prepare a sufficient volume to have 100 μL per assay; following that, add 5 μL Alexa Fluor*488 annexin V and 1 μL 100 μg/mL PI working solution to each 100 μL of cell suspension. After a period of about 15 minutes incubation in the room temperature, 400 μL 1X annexin-binding buffer was added into cells; next, the samples were gently mixed and kept on the ice. In the last stage, the stained cells were instantly analyzed by flow cytometry (BD, USA). All data were expressed as mean standard error of the mean unless noted. One-way analysis of variance with post hoc Tukey means comparison tests and unpaired Student’s t-tests were conducted with a significant level of p < 0.05. A minimum of three replicate samples were used for all experiments. 

## Results

**Figure 1:**
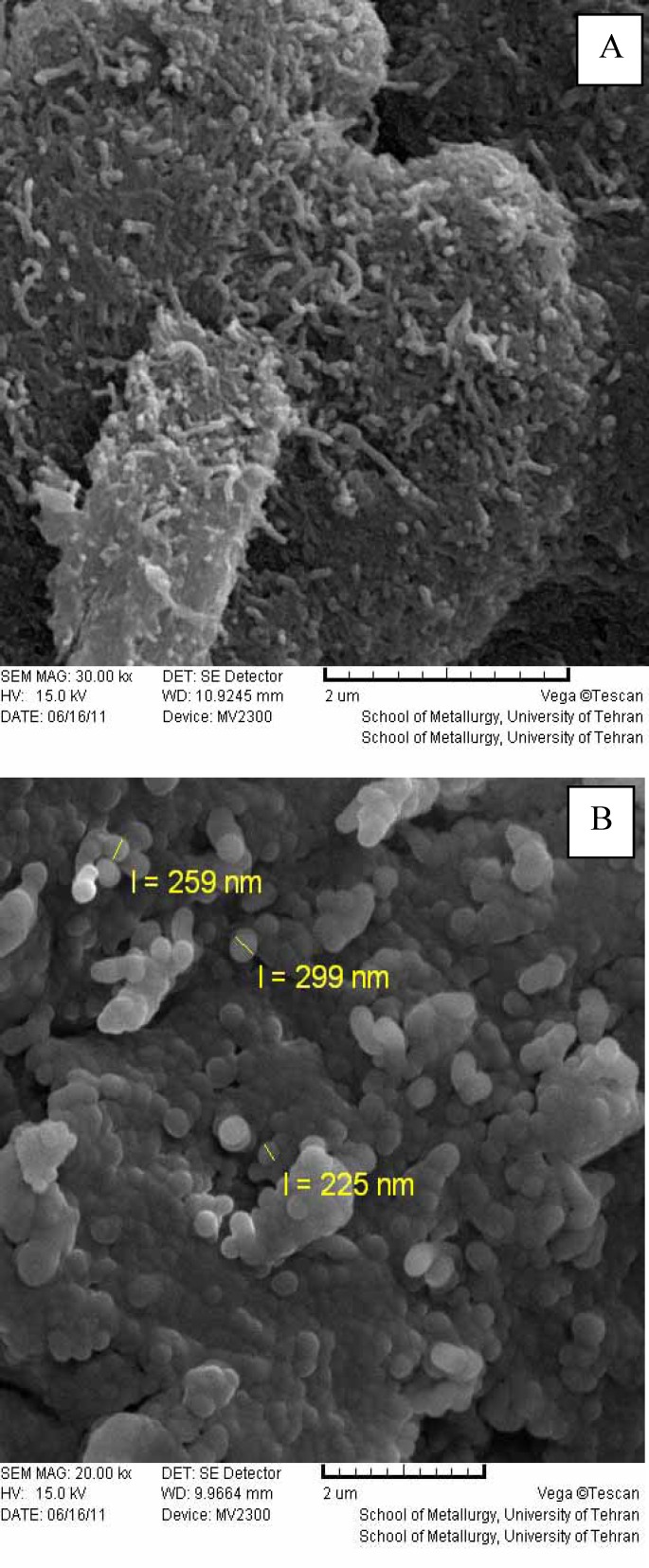
SEM images of non-conjugated MWCNTs (A), and conjugated CNTs with drug (B

 SEM images of carbon nanotubes showed that in the functionalized samples with PTX (B), a layer of uniform organic compounds is clear on the sidewall of the nano-tubes and diameters of samples are slightly increased (250 nm), compared to that without functionalizing (10 nm). These structures are quite different from those of the starting not functionalized carbon nanotube, in which the tube surface is relatively smooth and clean as depicted (Figure 1).

**Figure 2:**
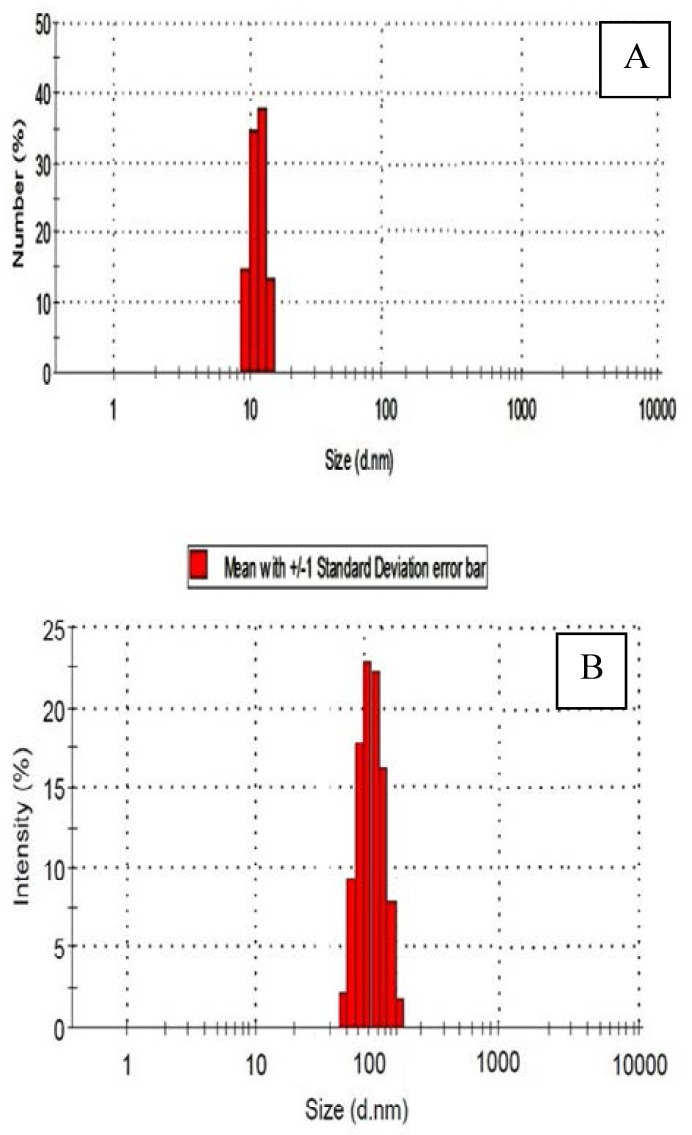
DLS diagram of carbon nanotubes. A): non-conjugated CNTs, and B) conjugated CNTs with paclitaxel drug

The size of the produced particles was analyzed with DLS, using the corresponding aqueous dispersions. Each sample was sonicated in aqueous solutions to the extent that shows reliable results (figure 2). An increase in the particle size of MWCNT from primary sample during the modification with the drug is readily observed and is consistent with our claim regarding preparation of complex. The average cluster size of carbon nanotubes is around 20 nm and this size for drug/carbon nanotubes is about 180 nm.

**Figure 3:**
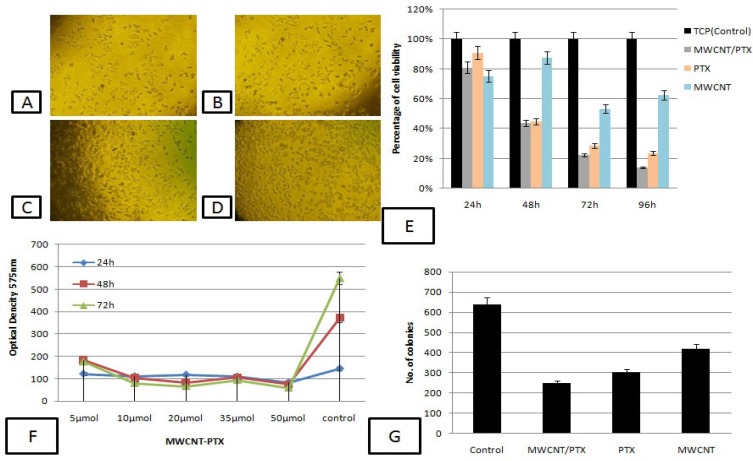
Invert microscopic images of samples with MKN-45 cells (A-D). A) Conjugated CNTs with paclitaxel drug (MWCNT/PTX), B) paclitaxel drug as a positive control, C) non-conjugated MWCNT and D) TCPS (tissue culture polystyrene) as a negative control. E) Cytotoxicity test by MTT assay in 24, 48, 72 and 96 h, F) IC50 calculation for conjugating CNTs with PTX, and G) CFU assay for conjugated CNTs with PTX (mean ± SEM , P < 0.05 Magnification×60 dates

**Figure 4:**
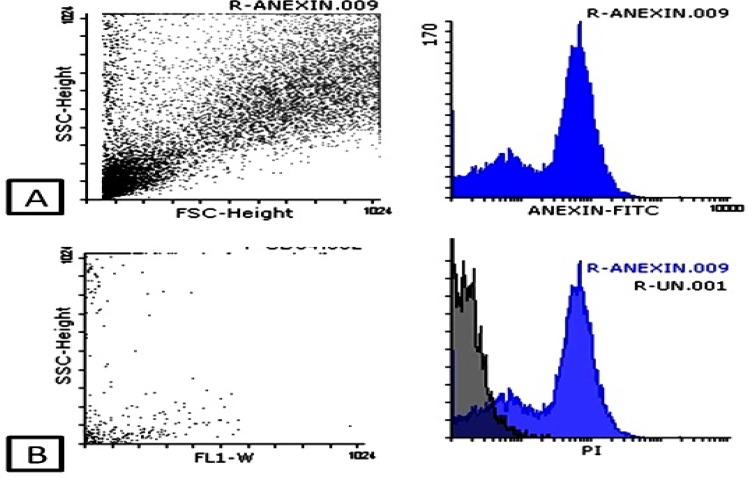
Apoptosis assay, analyze of annexin V by flowcytometry (Ex = 488 nm; Em = 350 nm) using FITC signal detector (A) and PI staining by the phycoerythrin emission signal detector (B

**Table 1:** Rate of necrosis and apoptosis on gastro adenocarcinoma cell line

Necrosis/ apoptosis	Necrosis (PI+/Anexin V-)	Late apoptosis (PI+/Anexin V+)	Apoptosis (PI-/Anexin V+)	Normal (PI-/Anexin V+)

Figure 3 shows MTT assay, colony forming unit assay (CFU), IC50 concentration for the TCPS (tissue culture polystyrene; negative control), paclitaxel as a positive control and un-conjugated and conjugated MWCNTs with PTX separately. Results have declared that a low and high viability for the PTX and TCPS are 20% and 100%, respectively. This viability decreased significantly for conjugated carbon nanotubes CNTs with paclitaxel drug compared to drug, as a result of which the reduction in viability (15%) for drug/CNTs complex sample could be attributed to the presence of carbon nanotubes groups. The presence of carbon nanotubes as a quite carrier can be the key point in such observations. Figure 3 (A-D) shows microscopic images of the cell culture on the prepared samples and control samples. Figure 3D reveals a good growth of the MKN-45 cells on TCPS and significant decrease in the number of cells in MWCNT/PTX (Figure 3A) and paclitaxel (Figure 3B) samples. Cuboidal-shaped cells in the vicinity of complex are observed in Figure 3 D. While for drug/CNTs complex sample and PTX, granular cells-shaped cells have been observed (Fig. 3 A, B). To calculate the IC50, concentration series for MWCNT / PTX were obtained and examined in cell culture system by MKN-45 gastro adenocarcinoma cell lines. The effective concentration of 20 µmol was reported as IC50 (Figure 3F). Colony-forming unit (CFU) assays provided a convenient means of assessing the clonogenic capacity of the MKN-45 cells expanded in culture. The clonogenic capacity of MKN-45 cells in TCPs group were 640 ± 4.8 (100%) colonies per 100 cells. Results for MWCNT/PTX have revealed that 250±3.6 (39%) colonies were formed per 100 cells by comparingf the clonogenic capacity of PTX and MWCNT were 305± 1.7 (48%), and 420± 3.2 (65.6%) colonies formed per 100 cells (p value=0.05). (Figure 3G). The apoptosis assay by flow-cytometry method was also performed to declare how MWCNT/PTXs effected on the ability of gastric cancer cell (Figure 4). Annexin V has a strong, Ca^2+^-dependent affinity for Phosphatidyl Serine and can be used as a probe for detecting apoptosis. The flow cytometry results have shown that MWCNT/PTXs might promote apoptosis in MKN-45 gastric adenocarcinoma cell line (Table 1). The necrosis and apoptosis toll on gastro adenocarcinoma cell line is: 3.04%, late apoptosis rate: 12.06%, apoptosis: 54.82%, and normal cell: 30.08%. 

## Discussion

According to the latest research, the inner and outer surfaces of pristine CNTs have various characteristics, offering the possibility of loading the inner space and outer surface with biologically active species to make it easier to use them in biological systems and improve their biocompatibility properties. Furthermore, bioactive agents can be conjugated to carbon nanotubes through functionalization, as a result of which they can serve as a suitable carrier for drugs, antigens and gene delivery. Also, through functionalization method, bioactive agents can diminish the toxicity of pristine CNTs (28-30). In another study, SWCNTs have been a pre-functionalized covalently with succinic anhydride-modified PTX, and folic acid (FA), as a targeting agent for many tumors toward cancer cells to open up new opportunities in chemical, biological, and medical applications of novel a nano-materials (19). In this approach both the FA and PTX covalently functionalized upon amide-SWCNTs. The FA and PTX were well-solubilized and stable in water, PBS, and cell medium containing fetal calf serum and full serum and biocompatibility and high targeting ability. Moreover, the covalently conjugated onto to modify SWCNTs were prepared by 1-(3-(dimethylamino) propyl)-3-ethylcarbodiimide hydrochloride (EDC) and N-hydroxysuccinimide (NHS) (19). It is suggested that the paclitaxel was successfully loaded onto the SWCNTs, which may be mainly broken up by covalent stacking and hydrophobic interactions. The concentration of paclitaxel loaded onto SWCNTs was measured by the absorbance peak at 240 nm with a molar extinction coefficient of 31.7 ×10^5^ M.cm^-1^. On the basis of optical absorbance data (UV-Vis-NIR spectra) of PTX and MWCNTs the quantity of PTX was measured, for the same batches of samples (19).

To investigate MWCNT loaded with PTX on proliferation of gastric cancer cells, it is used covalent method for drug loading or bonding then, MTT assay and clone formation test were scrutinized on MKN-45 cells. The MTT results have shown that the number of viable cells in MWCNT/ PTXs were significantly less than those in control sample at 24 h, 48 h, 72 h, and 96 h. While, the sample consists of PTX drugs have proved more viability compared to other groups (Figures 3). After that, cells were cultured for 14 days to perform the cloning formation assay. Despite the fact that both PTX and MWCNT/PTXs can decrease colony numbers compared to control groups, MWCNT/PTXs can decrease greater than 27% in the colony number compared to the drug alone group (Figure 3G). Cell cycles were blocked by PTX, MWCNT/PTXs and MWCNTs, compared to the control group (Figure 3G). The appearance of phosphatidylserine (PS) residues (normally hidden within the plasma membrane) on the cell surface is an early event in apoptosis, and can be used to detect and measure apoptosis. During apoptosis, PS is translocated from the cytoplasmic face of the plasma membrane to the cell surface. Annexin V has a strong, Ca^2+^-dependent affinity for PS and can be used as a probe for detecting apoptosis. Furthermore, apoptosis analysis assay was also performed to declare how MWCNT/PTXs effected on the ability of gastric cancer cell (Figure 4). The flow cytometry data were suggested that MWCNT/PTXs might promote apoptosis of MKN-45, as a result of which MWCNTs might partly enhance the effects of PTX on cell cycle or apoptosis compared with PTX alone.

In this study, PTX / MWCNTs complex was obtained using chemical methods. Microscopic analyses showed carbon nanotubes without physical changes after the conjugation process. Conjugation of organic combination with carbon nanotubes was confirmed using the data obtained from elemental analysis. In addition, the procedure developed in this study can be applied for cancer therapy.
